# In Situ Iodide Passivation Toward Efficient CsPbI_3_ Perovskite Quantum Dot Solar Cells

**DOI:** 10.1007/s40820-023-01134-1

**Published:** 2023-06-29

**Authors:** Junwei Shi, Ben Cohen-Kleinstein, Xuliang Zhang, Chenyu Zhao, Yong Zhang, Xufeng Ling, Junjun Guo, Doo-Hyun Ko, Baomin Xu, Jianyu Yuan, Wanli Ma

**Affiliations:** 1 Department of Materials Science and Engineering, Southern University of Science and Technology, Shenzhen, 518055 People’s Republic of China; 2 Institute of Functional Nano & Soft Materials (FUNSOM), Soochow University, 199 Ren-Ai Road, Suzhou Industrial Park, Suzhou, 215123 People’s Republic of China; 3 Department of Electrical and Computer Engineering, University of British Columbia, 2329 West Mall, Vancouver, BC V6T 1Z4 Canada; 4 Department of Chemistry, Sungkyunkwan University, Suwon, 16419 Republic of Korea; 5 Jiangsu Key Laboratory of Advanced Negative Carbon Technologies, Soochow University, 199 Ren-Ai Road, Suzhou Industrial Park, Suzhou, 215123 People’s Republic of China; 6 Jiangsu Key Laboratory for Carbon-Based Functional Materials & Devices, Soochow University, 199 Ren-Ai Road, Suzhou Industrial Park, Suzhou, 215123 People’s Republic of China

**Keywords:** CsPbI_3_ perovskite quantum dots, In situ passivation, Surface trap states, Perovskite solar cell

## Abstract

**Highlights:**

- The introduction of hydroiodic acid (HI) manipulates the dynamic conversion of PbI_2_ into highly coordinated species to optimize the nucleation and growth kinetics.
- The addition of HI enables the fabrication of CsPbI_3_ perovskite quantum dots with reduced defect density, enhanced crystallinity, higher phase purity, and near-unity photoluminescence quantum yield.
- The efficiency of CsPbI_3_ perovskite quantum dot solar cells was enhanced from 14.07% to 15.72% together with enhanced storage stability.

**Abstract:**

All-inorganic CsPbI_3_ quantum dots (QDs) have demonstrated promising potential in photovoltaic (PV) applications. However, these colloidal perovskites are vulnerable to the deterioration of surface trap states, leading to a degradation in efficiency and stability. To address these issues, a facile yet effective strategy of introducing hydroiodic acid (HI) into the synthesis procedure is established to achieve high-quality QDs and devices. Through an in-depth experimental analysis, the introduction of HI was found to convert PbI_2_ into highly coordinated [PbI_m_]^2−m^, enabling control of the nucleation numbers and growth kinetics. Combined optical and structural investigations illustrate that such a synthesis technique is beneficial for achieving enhanced crystallinity and a reduced density of crystallographic defects. Finally, the effect of HI is further reflected on the PV performance. The optimal device demonstrated a significantly improved power conversion efficiency of 15.72% along with enhanced storage stability. This technique illuminates a novel and simple methodology to regulate the formed species during synthesis, shedding light on further understanding solar cell performance, and aiding the design of future novel synthesis protocols for high-performance optoelectronic devices. 
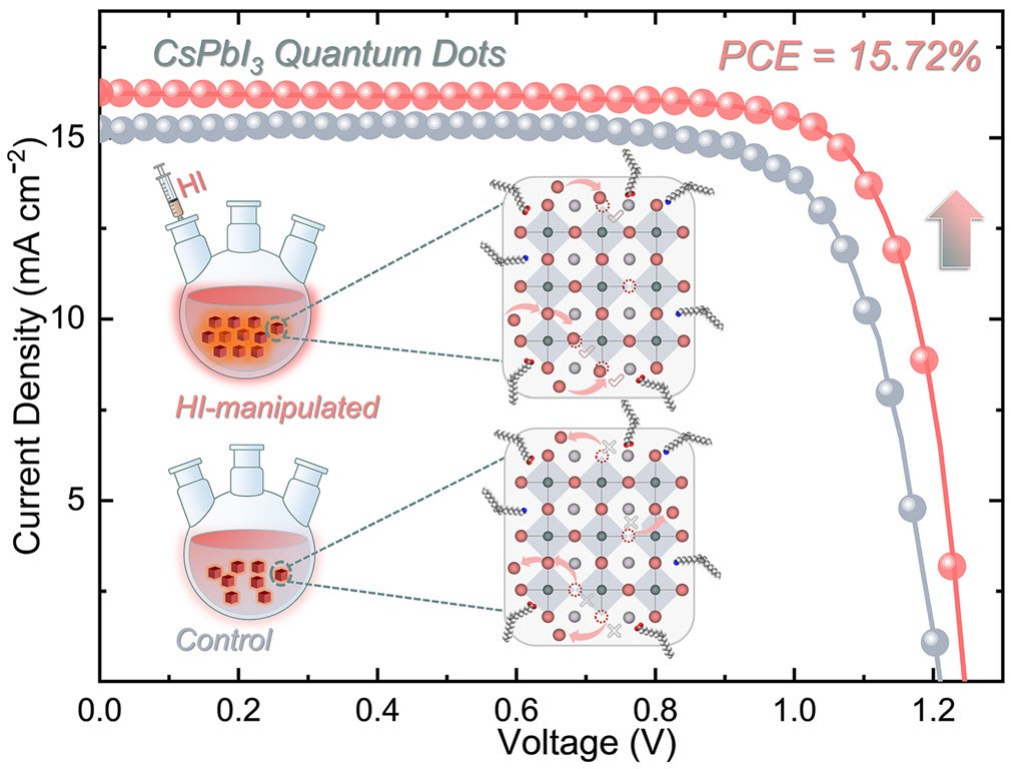

**Supplementary Information:**

The online version contains supplementary material available at 10.1007/s40820-023-01134-1.

## Introduction

Solution-processed all-inorganic lead halides colloidal perovskite quantum dots (QDs) have been extensively studied as photo-electron conversion and emitting materials for optoelectronic applications [1-5]. They exhibit superior optical properties, such as a spectrally tunable bandgap, narrow emission width, and high photoluminescence quantum yield (PLQY) [5, 6]. Blessed with excellent properties, perovskite QDs quickly opened a new horizon in the optoelectronic field [7-9]. Perovskite QDs with inorganic cations, such as CsPbI_3_, have recently gained increasing attention due to superior ability in controlling size, shape and composition, as well as possessing a desired solar-cell optical bandgap of 1.73 eV [1, 10, 11]. Additionally, it was demonstrated that CsPbI_3_ QDs can maintain a stable cubic (black) phase at room temperature, whereas bulk CsPbI_3_ materials tend to convert into their nonfunctional δ-phase (yellow) below a temperature of 320 °C [6, 12, 13]. These non-functional crystals demonstrate poor optoelectronic properties [14]. The superior phase stability in CsPbI_3_ QDs is mainly accredited to the contributions of its high surface-to-volume ratio and attached capping ligands which sterically insulate the QDs from environmental damage [15-18].

While surface ligands ensure phase stability, their inherent insulating nature inevitably hinders charge transport [19, 20]. Layer-by-layer device deposition allows for the chemical removal of surface ligands. While this in theory improves charge transport along the semiconductor, it inescapably generates dangling bonds which act as trap states, impeding carrier diffusion and transport [10]. Surface post-processing is considered one of the most effective methods to eliminate unnecessary recombination centers generated from these ionic vacancy defects [17, 21]. Quite recently, great research efforts are concentrated on functional grouped organic molecules such as triphenyl phosphite [22], di-n-propylamine [20], GA^+^ salts [23], and phenylethyl ammonium to minimize surface point defects induced recombination centers and enhance the dot-to-dot electronic coupling [24]. Moreover, significant previous work on perovskite QDs and PbX (S, Se) QDs has shown that metal cations and inorganic ions can modulate the surface dangling bonds of QDs to promote charge transport [19, 25]. Solid-state post-processing passivation strategies for achieving lower surface defect state densities are mainly focused on the surface of the CsPbI_3_ QD layer [26, 27]. However, trap states underneath remain insufficiently passivated. Additionally, based on the established colloidal synthetic protocols, PbI_2_ serves as the sole source of iodide ions, which leads to an excessively high demand for PbI_2_ within the crystal [28]. Such high Pb-rich requirements render a significant portion of unreacted Pb and Pb-related byproducts [29, 30]. Therefore, developing a simple and effective in situ strategy for minimizing the trap state density in QDs while reducing lead waste is of great significance.

In this work, we employ an in situ passivation method by introducing hydroiodic acid (HI) into the precursor solution to obtain high-quality CsPbI_3_ QDs. We systematically tuned the added concentration of HI and characterized the structural, optical, electrical, and morphological properties. The deployment of the in situ passivation strategy was found to not only enhance the crystallinity but also lead to a reduced defect density. These improvements stem from the HI-driven conversion of the uncoordinated Pb^2+^ion into [PbI_m_]^2−m^. A suitable iodine ion introduction can guarantee the fabrication of a CsPbI_3_ QD matrix with decreased non-radiative recombination caused by iodine-vacancy point defects, forming compact low defect-density polycrystalline QD films [31-33]. Consequently, this passivation technique yielded a best power conversion efficiency (PCE) of 15.72% together with enhanced storage stability.

## Experimental

### Materials

1-octadecene (ODE, tech. grade, 90%, J&K), Cs_2_CO_3_ (99.9%, J&K), oleic acid (OA, tech, grade, 90%, Alfa), oleylamine (OLA, tech. grade, 90%, Alfa), lead iodine (PbI_2_, 99.0%, Advanced Election Tech.), titanium tetrachloride (TiCl_4_, ≥ 98%, Sinopharm Chemical Reagent Co., Ltd.), n-hexane (> 98%, Alfa Aesar), methyl acetate (MeOAc, anhydrous 99.5%, Sigma), hydroiodic acid (HI, 95%, Sigma), 1-Octane (anhydrous, 99.8%, Sigma), Poly[bis(4-phenyl)(2,4,6-trimethylphenyl)amine]: PTAA (Mn = 17,000 g moL^−1^, Xi’an Polymer Light Technology Corp) were purchased and used as received without further purification.

### Synthesis and Purification of CsPbI_3_ QDs

PbI_2_ (1 g) and ODE (50 mL) were added into a 250 mL round bottom three-neck flask. The solution is then slowly heated to 90 °C under vacuum for at least one hour. Then, the flask is filled with nitrogen (N_2(g)_) and injected with 5 mL of both OA and OLA. The flask is then put again under vacuum and subsequently filled with N_2_. Next, the solution is slowly heated to 165 °C. As this temperature stabilizes, the preheated transparent Cs-oleate (8 mL) is swiftly injected into the Pb-I-precursor and allowed to react for a reaction time of 5 s. The solution is then quickly cooled by using an ice-water bath. In the HI-manipulated CsPbI_3_ QDs synthesis, different feeding volumes of HI solution (50, 100, and 150 μL) were loaded into the PbI_2_-precursor, with an identical remaining procedure. For purification, the crude solution of CsPbI_3_ QDs was precipitated by adding MeOAc (the volume ratio of methyl acetate (MeOAc) and the as-synthesized solution is 3:1), and the mixture was centrifuged at 8000 rpm for 5 min. The supernatant was discarded, and precipitates were redispersed in 18 mL of hexane. Then, the solution was mixed with 18 mL MeOAc and centrifuged at 8000 rpm for 3 min. The supernatant was discarded, and the received precipitate was redispersed in 20 mL hexane. Finally, the solution was centrifuged at 4000 rpm for 5 min to remove large aggregates, while the supernatant was collected. The obtained supernatant was stored at − 5 °C for 24 h in a dark condition and centrifuged again at 4000 rpm for 5 min to precipitate byproducts and unreacted materials. The final precipitate was dried through a rotary evaporator and redispersed into octane with a concentration of 70 mg mL^−1^.

### CsPbI_3_ QD Solar Cell Fabrication and Characterizations

CsPbI_3_ QD solar cells were constructed with a structure of glass/fluorine-doped tin dioxide: (FTO)/TiO_2_/CsPbI_3_QDs/Poly[bis(4-phenyl)(2,4,6-trimethylphenyl)amine]:(PTAA)/MoO_3_/Ag. FTO substrates were ultrasonically cleaned in deionized water, acetone, and isopropanol several times. Next, compact TiO_2_ films were deposited onto the cleaned FTO substrates via chemical bath deposition at 70 °C [34]. The films were annealed at 200 °C for 30 min and then, further treated with UV-ozone for 20 min. The CsPbI_3_ QD solution (70 mg mL^−1^ in octane) was spin-casted on the substrate at 1000 rpm for 20 s and 2000 rpm for 15 s. Then, 120 µL of methyl acetate (MeOAc) was dropped on the as-casted CsPbI_3_ QDs layer for 5 s to ignite the solid ligand exchange process to remove the long chain ligands, followed with a final centrifugation at 2000 rpm for 20 s. This fabrication process was repeated four times to establish a thick QD film of ~ 400 nm to provide sufficient light absorption. Then, the film was immersed into the solution of Guanidine thiocyanate (GASCN) in ethyl acetate (EtOAc) followed by a rinse and drying in MeOAc and N_2_, respectively. The CsPbI_3_ QD film fabrication process was conducted in a dry air-filled glove box at room temperature with relative humidity below 10%. The doped PTAA toluene solution (15 mg mL^−1^) was spin-coated on top of the CsPbI_3_ QD films at 3000 rpm for 40 s. Finally, 8- and 120-nm-thick layers of MoO_3_ and Ag were deposited by thermal evaporation under a vacuum of 1 × 10^−6^ mbar, respectively. The active area of the cell was defined as 0.0725 cm^−2^ through a shadow mask. The *J–V* characteristics of the devices were acquired using a Keithley 2400 digital source meter under simulated air-mass 1.5G (AM 1.5 G) spectrum at 100 mW cm^−2^ with a solar simulator (Class AAA, 94023 A-U, Newport). The light intensity was calibrated to 100 mW cm^−2^ by a National Renewable Energy Laboratory certified monocrystalline silicon reference solar cell (91 150 V, Newport Oriel).

## Results and Discussion

### Investigation of Optical and Carrier Dynamics Properties

**Fig. 1:**
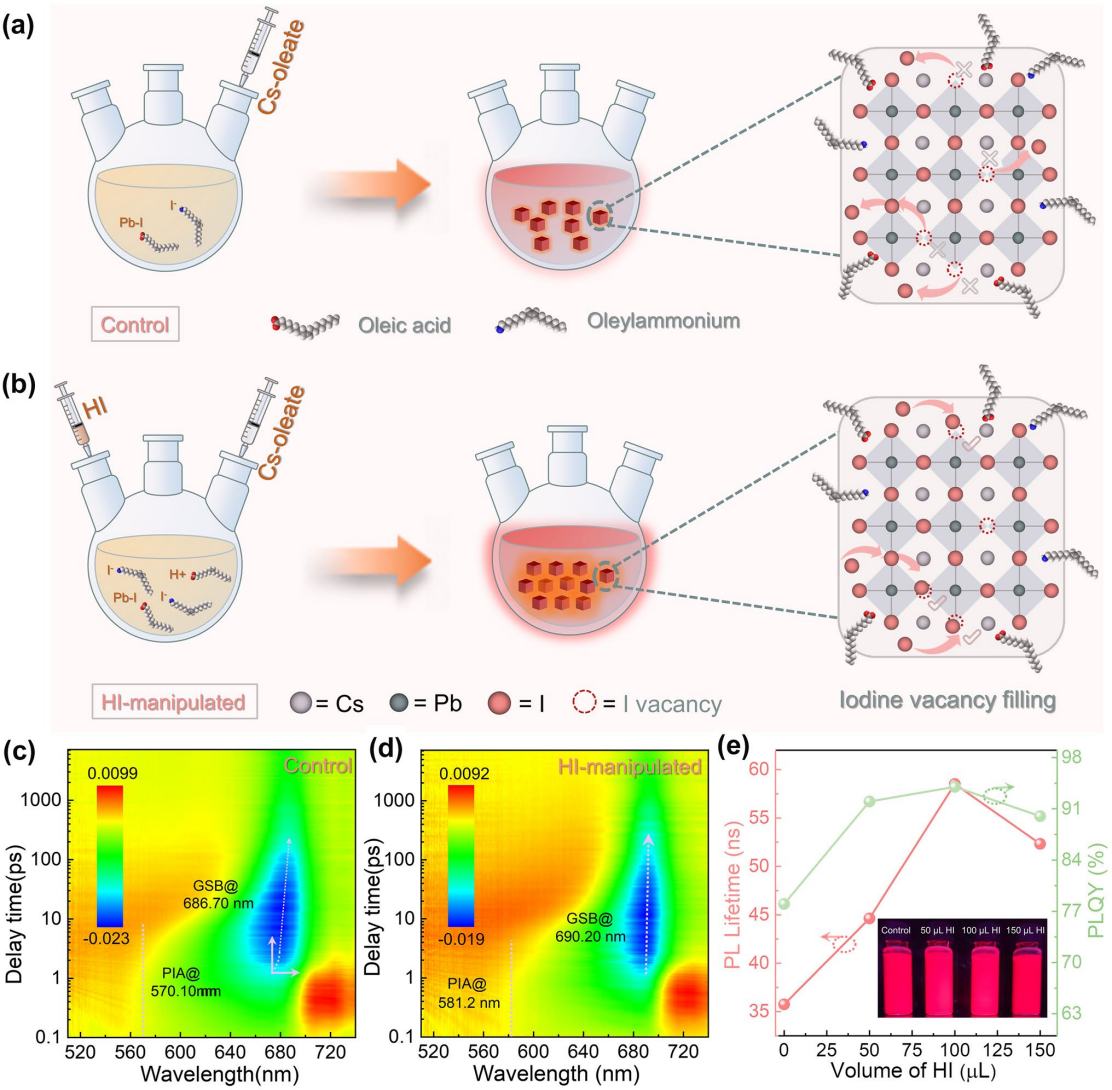
**a, b** Schematic illustration of CsPbI_3_ QDs synthesis process w/wo HI manipulation. **c, d** Transient absorption maps of control and HI-manipulated CsPbI_3_ QDs. and **e** PLQY and the fitted TRPL lifetimes of CsPbI3 QDs synthesized with different HI volumes (Inset: photographs of QD solutions under UV light illumination)

The colloidal CsPbI_3_ QDs were synthesized via the reported hot-injection protocol using oleic acid (OA) and oleylammonium (OLA) ligands [35]. The HI-manipulated CsPbI_3_ QDs utilized in this research were synthesized by first injecting vary volumes of HI into PbI_2_-precursor, followed by the injection of Cs-oleate, as illustrated in Fig. 1a, b. We investigated the UV–vis absorbance and steady-state photoluminescence (PL) spectra of the CsPbI_3_ QD solution with/without (w/wo) HI-manipulation. For HI-modified QDs, the PL emission peaks display a gradual red shift from 685 to 691 nm for control and QDs with HI manipulation below 100 μL, except for the 150 μL HI sample, which exhibits opposite trends (Fig. S1**)**. The optical bandgaps were extracted from the Tauc plots in Fig. S2 with a value of 1.785 (± 0.005) and 1.776 (± 0.003) eV for the control and optimal HI devices, respectively. It is noted that HI manipulation exhibits a negligible impact on the optical absorption (Fig. S3), while creating an emission shoulder near the main PL peak position at 688 nm for the 150 μL sample (Fig. S1). This emission shoulder is attributed to the emergence of nanowires throughout the crystal morphology. To examine the passivation effect of HI on CsPbI_3_ QDs, we performed time-resolved photoluminescence (TRPL), PL quantum yield (PLQY), 2D-PL, and transient absorption spectra (TAS) measurements. Figure 1c, d depicts the 2D TAS patterns for CsPbI_3_ QDs w/wo HI. The positions of the photo-bleaching peak at 5 ps in the initial signal emergence are well-aligned with the exciton peaks from steady-state absorption. The photo-excited carriers then rapidly funnel to the low-energy sites [36]. While the HI-manipulated sample exhibits minor energy offset, the control sample exhibits a gradually redshift of the photo-bleaching peak characteristic (Fig. S5a–b). The reduced surface iodine-vacancy-induced lattice disorder is attributed to the lowered energy offset, which indicates less disorder and energy funneling toward undesired band-tail states [37]. The derived decay curves of ground states bleaching and photon-induced absorption from TAS spectra are displayed in Fig. S5c–d, and Table S1provides a detailed list of the fitted parameters. As shown in Figs. S4 and 1e, the observed TRPL results exhibit a bi-exponential decay characteristic, and the fitted average PL lifetimes (*τ*_avg_) are 35.74 and 58.51 ns for the samples w/wo HI passivation (Table S2), respectively. This prolonged exciton recombination dynamic implies the successful suppression of surface trap-induced nonradiative recombination [36, 38, 39]. In addition, the optimal HI dosed QDs observed a near-unity PLQY of 94%, whereas the control is merely 78% (Fig. 1d, Table S3), indicating excellent surface passivation [40]. These results further demonstrate that the addition of HI aids in obtaining CsPbI_3_ QDs with lower crystallographic defects. These observations are in good agreement with the results of TA characterizations.

### Crystallization and Morphology Examination of CsPbI_3_ QDs

**Fig. 2:**
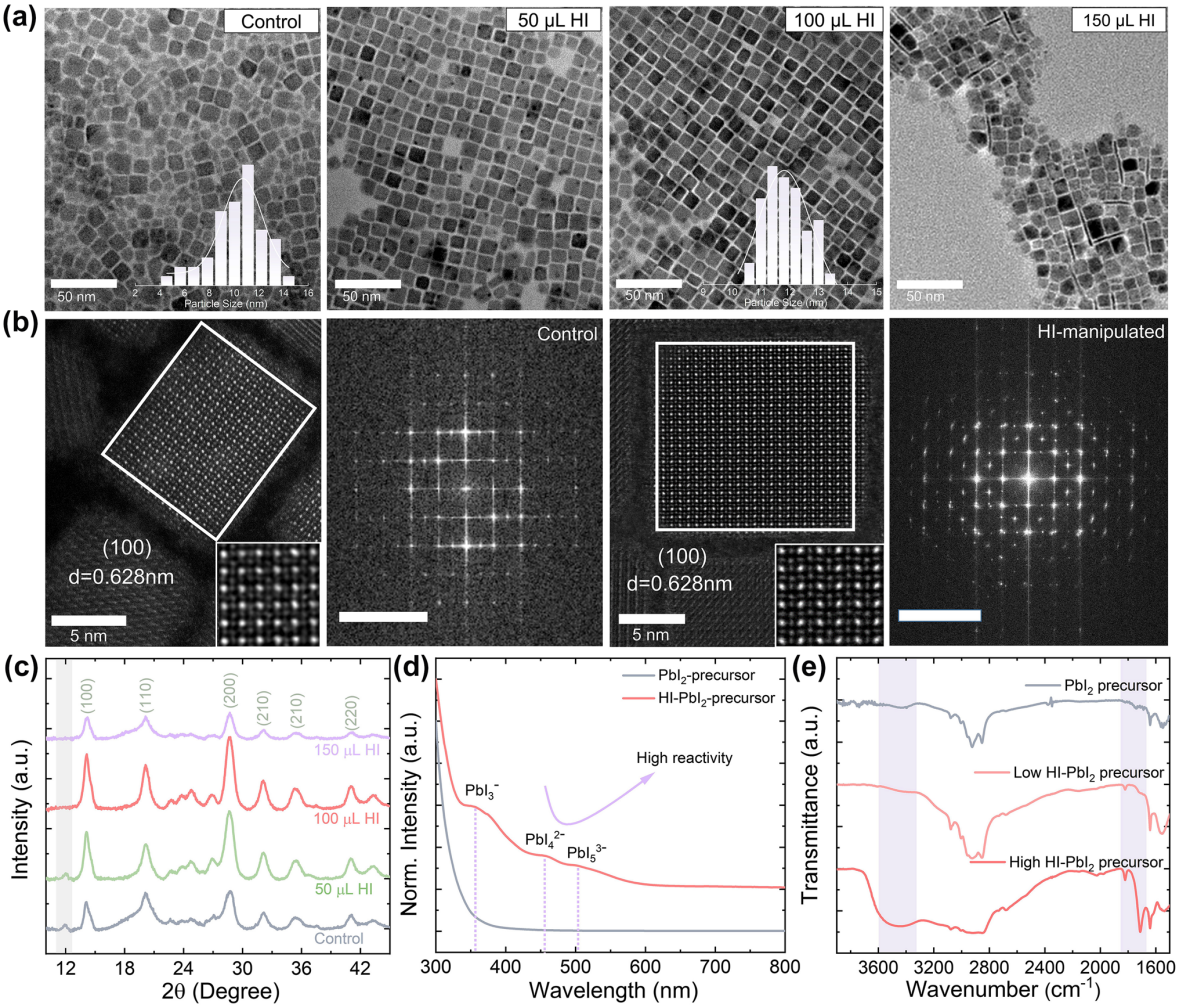
**a** TEM images of the CsPbI_3_ QDs synthesized with varying HI concentrations (Inset: the statistic distribution of grain sizes extracted from the corresponded TEM images). **b** The spherical aberration corrected TEM images of the control and optimized HI-manipulated CsPbI_3_ QDs, along with the corresponding FFT patterns (Inset: high-magnification TEM images shown the lattice distance of 0.628 nm of (100) crystallographic plane). **c** Evolution of XRD patterns of the CsPbI_3_ QDs synthesized with different HI volumes. **d** UV–vis absorption spectra and **e** FTIR spectra of PbI_2_-OA-OLA precursor w/wo the addition of HI

To reveal the effects on the crystal morphology, a set of transmission electron microscope (TEM) images were acquired. As shown in Fig. 2a, the deployment of the HI assisted in situ synthetic procedure did not change the morphology below 100 μL. Surpassing this volume threshold leads to the emergence of nanowires (Figs. 2a and S1, S6). Good size uniformity is crucial for charge carrier non-radiative recombination as a wide size distribution causes the broadening of the band-tail states which aggravates the energetic disorder [41, 42]. To analyze this, TEM images were obtained and the QD size distribution was examined. As shown in Fig. 2a (the insets), the histograms are fitted using a Gaussian curve to determine the average size. The mean particle size of the control and optimal HI-manipulated CsPbI_3_ QDs are 10.25 and 11.84 nm, respectively. The results indicate that a significant improvement in the size uniformity, along with relatively larger QDs size. In addition, structural characterizations were also conducted. Spherical aberration corrected TEM was used to observe the atomic structures and characterize the crystallinity of the CsPbI_3_ QDs, as shown in Fig. 2b. Both the control and HI-influenced CsPbI_3_ QDs have a lattice separation of 0.628 nm, corresponding to the (100) crystal facet of the cubic structure [1, 43, 44]. Using these TEM images, the crystalline structures were analyzed by measuring the fast Fourier transformation (FFT) (Fig. 2b) and compared with the X-ray diffraction (XRD) patterns (Fig. 2c). These results indicate an ideal cubic phase along with an unmodified zone axis after the introduction of HI [45]. Moreover, as shown in Fig. 2c, the XRD patterns suggest that the crystal structures are unaltered after HI addition. There is a notable peak of PbI_2_ at roughly 11.8° in the control sample, pointing to the presence of residual unreacted Pb-related byproducts [28, 46, 47]; however, the PbI_2_ diffraction peak disappears for samples with a feeding volume exceeding 50 μL. The diffraction peaks can be well indexed to the pure cubic CsPbI_3_ perovskite crystal structure, which is in good agreement with the standard data. Notably, the XRD diffraction intensity becomes stronger as the HI volume was kept below 100 μL. Surpassing this threshold leads to a substantial intensity decrease, while the cubic crystal structure is still retained. The XRD patterns of CsPbI_3_ QDs are consistent with the spherical aberration corrected TEM measurements, illuminating the improved QD crystallinity. To verify the morphological variation, we further investigate the results after overloading the HI additive. Like above, the optical and structural properties of the CsPbI_3_ QDs with overloaded HI manipulation were studied by measuring the UV–vis absorption spectra and TEM imaging. Interestingly, the absorption remains relatively unchanged (Fig. S7). However, via TEM imaging, we notice the formation of large aggregate crystals with irregular shapes and significantly increased nucleation sites (Fig. S8). Fundamental understanding into the chemical nature of nucleate numbers and growth process of CsPbI_3_ QDs can be discovered through these HI additives to promote the generation of polyiodide colloids. The iodine anions functioning as a Lewis base can interact strongly with halogenated metal molecules through noncovalent interactions, resulting in halogen bonding [48]. Also, UV–vis, XRD, and FTIR characterizations of the PbI_2_ and HI-PbI_2_-precursors were carried out to determine how the introduced acid impacts the crystallographic growth kinetics. Several iodide-coordinated plumbate ions are observed in the HI-PbI_2_-precursor, as shown in Fig. 2d, and their absorption peaks are consistent with the previous observations [49, 50]. We found that the high I^−^ concentration can trigger the generation of lead acid species including PbI_3_^−^ to PbI_4_^2−^ and PbI_5_^3−^, which indicates that I^−^ cooperates with the lead Lewis acid species to form highly coordinated polyiodide colloids. The XRD pattern seen in Fig. S9a further supports similar findings. The potential dynamics process of existing specials in Pb-I-precursor w/wo HI manipulation seen in Fig. S10. As shown in Fig. 2e, in the PbI_2_-precursor without HI, the stretching vibration of R-NH_3_^+^ is at 3433 cm^−1^ due to the protonation interaction with OA [20]. After the addition of HI, the stretching vibration is intensified and red-shifted to 3443 cm^−1^ due to the promoted protonation process and increased coordination of I^−^ with the PbI_m_^[2−m]^ colloids. The FTIR results further demonstrate that I^−^ works well with the lead acid species to form high-coordination centers. Since the reactivity of [PbI_m_]^2−m^ increases with the coordinate number m, PbI_4_^2−^ and PbI_5_^3−^ display have higher reactivity than PbI_3_^−^ [50]. Moreover, the additionally introduced HI raised the critical concentration of monomer, as shown in Fig. S9b**.** It is therefore plausible to conclude that the highly coordinated [PbI_m_]^2−m^ induced by HI introduction can surpass the limit of the reaction barrier to form CsPbI_3_ QDs, and thereby well manipulates the nucleate number and growth kinetics of CsPbI_3_ QDs, leading to enhanced crystallinity and phase purity.

### Analysis of CsPbI_3_ QD Film Variations

**Fig. 3:**
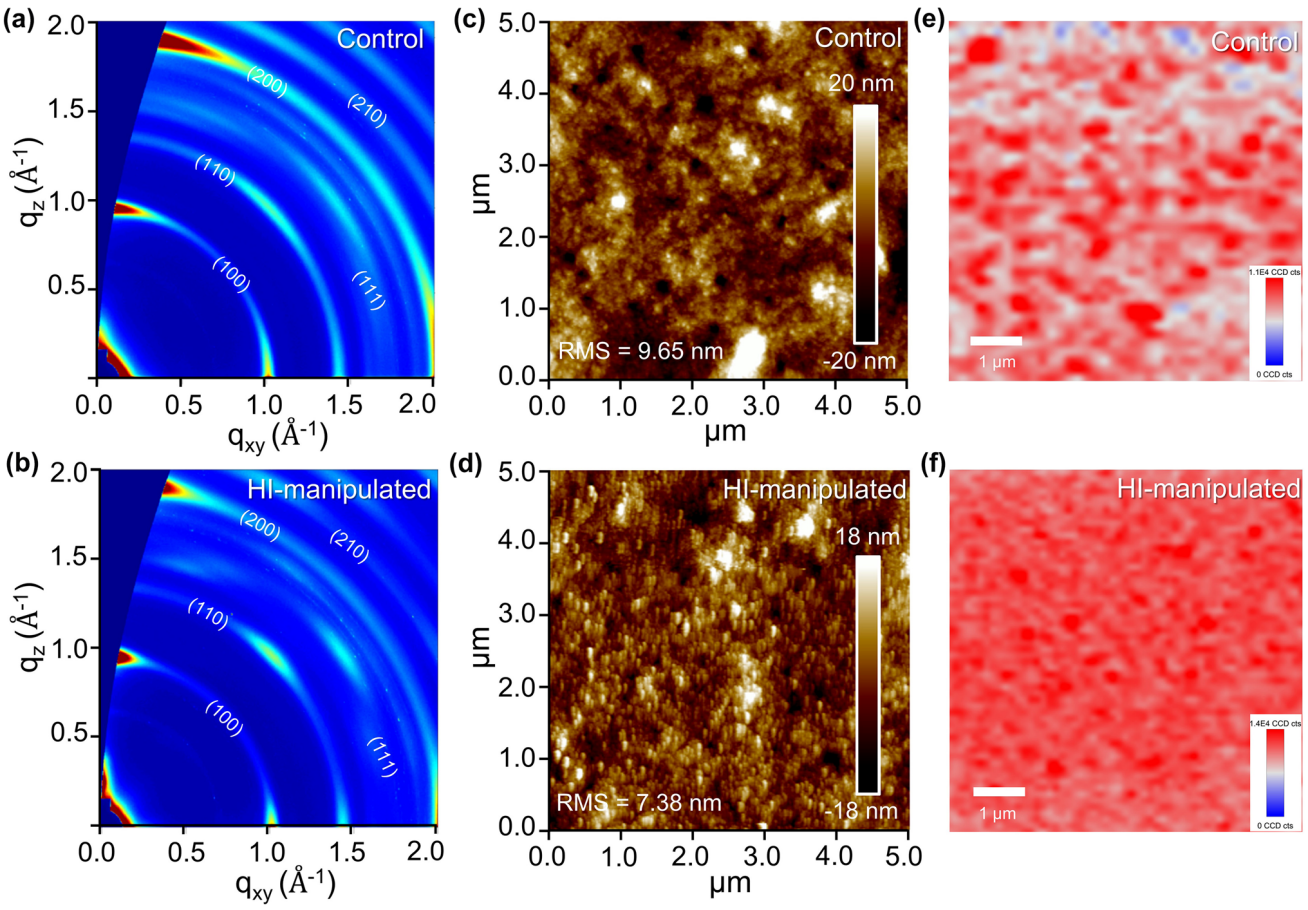
The control and optimal HI manipulated CsPbI_3_ QD films measurements of **a, b** 2D grazing incidence wide-angle X-ray scattering (GIWAXS) patterns, **c, d** Top-view atomic force microscopy (AFM) images and **e, f** 2D photoluminescence mapping

To gain insight into variations of the CsPbI_3_ QD films w/wo HI, grazing incidence wide-angle X-ray scattering (GIWAXS), top-view atomic force microscopy (AFM) and 2D-photoluminescence (2D-PL) mapping were further employed. The crystallinity and orientation preference of CsPbI_3_ QDs films were evaluated by synchrotron-based 2D-GIWAXS, as shown in Fig. 3a, b. Both samples display strong characteristic X-ray diffractions for CsPbI_3_ QDs. The 2D-GIWAXS diffraction pattern of the control sample exhibits diffraction intensity scattered around rings, indicating randomly orientation. In contrast, the HI manipulated film shows more localized diffractions peaks, suggesting preferable orientation. To examine the preferred direction of the crystallization process, we analyzed the extracted in-plane (*q*_*xy*_) diffractions peak curves. The diffraction patterns at 1.015, 1.429, 1.895, 2.029, and 2.258 Å correspond to the (100), (110), (111), (200), and (210) crystal planes for the typical CsPbI_3_ QDs structure, respectively [51]. We observed that the intensity of (100) lattice plane peak exhibits an evidently stronger in HI-manipulated QD sample, whereas the (200) remains consistent with the control (Fig. S11). As shown in Fig. 3c, d, both films exhibit a closely packed QD matrix with similar surface topography and roughness, confirming that the optimal volume of HI has a negligible impact on the CsPbI_3_ QD film. In addition, we performed 2D-PL mapping measurements of CsPbI_3_ QD films (Fig. 3e, f). The HI-manipulated films display an enhanced and more uniform PL emission intensity relative to the control. These results indicate that the introduced HI provides huge potential in controlling the crystal growth to acquire superior crystallinity of CsPbI_3_ QDs. To gain insight into the films surface chemistry variations, we performed X-ray photoelectron spectroscopy (XPS). The XPS core-level spectra of the constituent elements are shown in Fig. S12a–d. The bonding states corresponding to the Cs *3d*, Pb *4f*, and I *3d* core levels of the HI-manipulated CsPbI_3_ QDs shift marginally to higher binding energy regions with respect to the control, which can be attributed to the enhanced chemical interaction between surface lead and iodine ions. For CsPbI_3_ QDs synthesized with the HI, the ratio of I_3_/Pb is increased, and values of 1.56 and 1.79 are calculated for the control and HI-manipulated CsPbI_3_ QDs, respectively (Fig. S13). These results show that the CsPbI_3_ QDs was successfully restored by filling in the iodide vacancies, resulting in improved optoelectronic properties of the CsPbI_3_ QDs [18, 31].

### Photovoltaic Performance of CsPbI_3_ QD Solar Cells

**Fig. 4:**
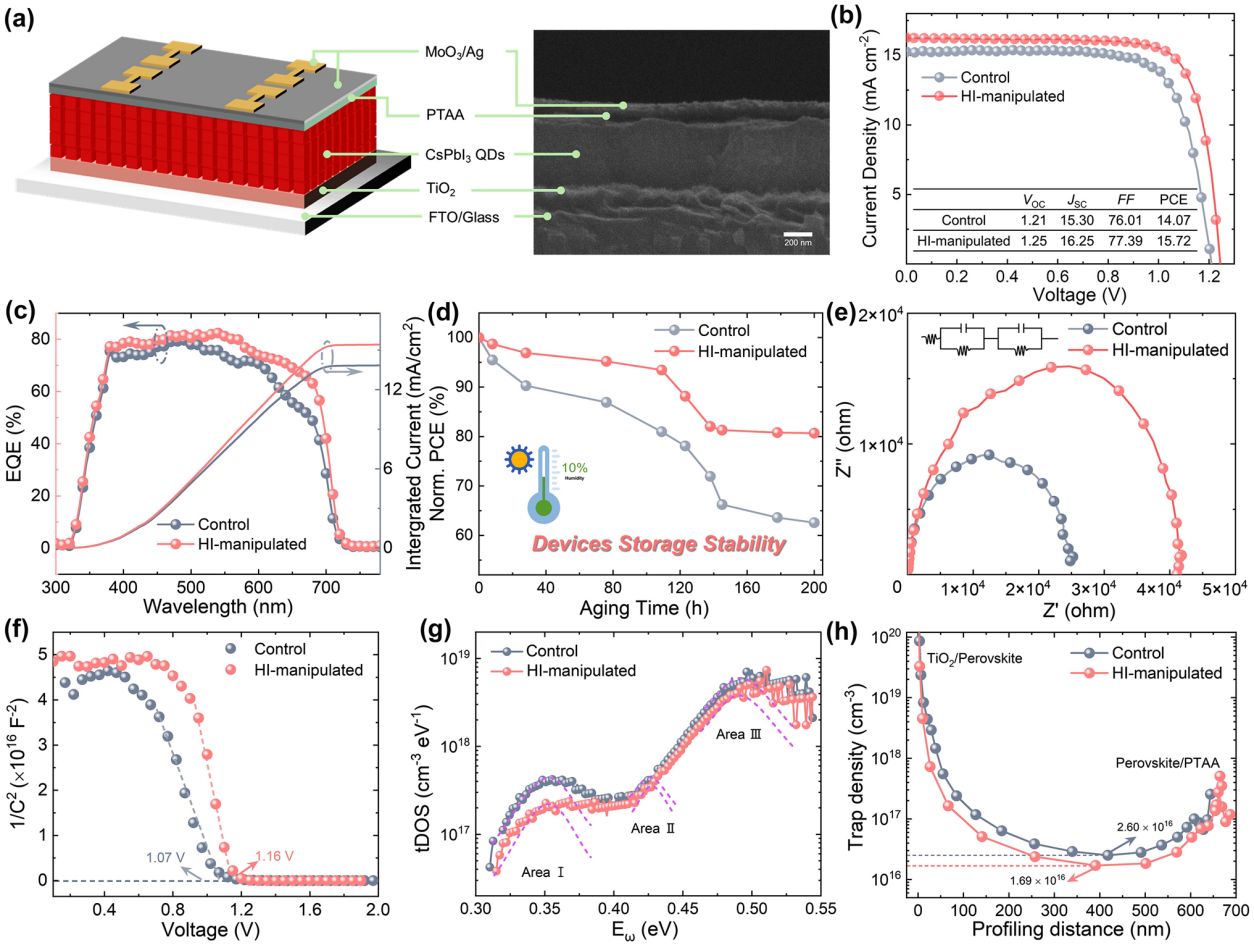
**a** Schematic illustration of device structure and the corresponding cross-sectional SEM image of CsPbI_3_ QD solar cell. **b** *J*–*V* curves of the devices based on control and 100 μL HI-manipulated CsPbI_3_ QDs. Inset: The detailed devices parameters of champion solar cells. **c** EQE spectra and integrated current density of optimized CsPbI_3_ QD solar cells. **d** Evolution of the PCE of the optimized CsPbI_3_ QD solar cells in dry air conditions. **e** Nyquist plots, **f** Mott–Schottky plots, **g** Trap density of states (tDOS) and **h** dependence of the trap densities on the profiling distances of optimized devices based on control and 100 μL HI-manipulated CsPbI_3_ QDs

Having examined the effective defects passivation of HI-manipulation on CsPbI_3_ QDs, it is important to explore whether the regulations can be combined and reflected on the performance of PV device. As mentioned above, the solar cells were fabrication with the following configuration: glass/FTO/TiO_2_/CsPbI_3_ QDs/PTAA/MoO_3_/Ag. A cross-sectional SEM view of the device is shown in Fig. 4a. CsPbI_3_ QD solar cells were fabricated by employing CsPbI_3_ QDs w/wo HI additive. Devices using HI volumes of 50, 100 and 150 μL were investigated with an optimal device performance achieved at 100 µL (Fig. S14**)**. Consequently, this volume, denoted as HI-manipulated, is the additive feeding volume used in all studies hereafter described. The *J–V* characteristics (under AM 1.5 G illumination with light intensity of 100 mW cm^−2^) of the control and HI-manipulated CsPbI_3_ QD solar cells are shown in Fig. 4b. We obtained the HI-manipulated device with a champion efficiency of 15.72%, an open-circuit voltage (*V*_OC_) as high as 1.25 V, a short-circuit current density (*J*_SC_) of 16.25 mA cm^−2^, and a fill factor (FF) of 77.39%. For the control, an output of 14.07% is achieved. The *J–V* curves under the forward scan direction are shown in Fig. S15. Detailed PV parameters extracted from the *J*–*V* curves in both forward and reverse scan directions are shown in Table S4. It should be noted that the HI-manipulated CsPbI_3_ QD solar cell demonstrates negligible hysteresis compared with the control device, which attributed to the effective surface iodine vacancies filling, leading to reduced ion migration. The improved passivation effect is unimpaired to reflect on the final PV outcome. The external quantum efficiency (EQE) as a function of wavelength for the solar cells is shown in Fig. 4c. As expected, the spectral response of the HI-manipulated device in the 380–700 nm wavelength range is superior to that of the control, which contributes to the improved *J*_SC_. The integrated current density from the EQE is 15.42 mA cm^−2^, which is in good agreement with the observed *J*_SC_ from the *J–V* characteristics. To explore whether the enhanced crystallinity will affect stability, the storage stability of the HI-manipulated CsPbI_3_ QDs was recorded. After storage under dry air conditions at room temperature for 200 h, the HI-manipulated CsPbI_3_ QD device retained 80.68% of its original PCE, while the control device showed a 37.43% PCE loss (Fig. 4d). The lattice deformation of the Pb-centered octahedral framework, which may be triggered by ion migration, causes the symmetry lowering. The in situ HI-manipulated CsPbI_3_ quantum dots were effectively restored by filling in the iodide vacancies, which led to the decreased ion migration, aiding to the reduced hysteresis and improved storage stability. In light of this, we attribute the notably improved storage stability to the effective passivated surface traps. To investigate the device charge recombination dynamic behaviors, electrochemical impedance spectroscopy (EIS) measurements were employed. The extracted parameters of the equivalent circuit are listed in Table S5. As shown in Fig. 4e, the series resistance (*R*_s_) and increased recombination resistance (*R*_rec_) of the HI-manipulated CsPbI_3_ QD devices indicate that the recombination process is effectively suppressed, resulting in improved charge transfer [52]. We further characterized the built-in potentials (*V*_bi_) of the two devices by using Mott–Schottky (M–S) analysis, as shown in Fig. 4f. The relationship between the junction capacitance and DC voltage bias can be described by the following equation [53]:

$$\frac{{A^{2} }}{{C^{2} }} = \frac{{2\left( {V_{{{\text{bi}}}} - V} \right)}}{{qN\varepsilon \varepsilon_{0} }}$$

where *A* is the active area of the device, *V* refers to the applied DC voltage, *q* is the elementary charge, *N* refers to the impurity doping density, and lastly, *ε* and *ε*_0_ refer to vacuum and relative permittivity, respectively. The *V*_bi_ can be calculated using the x-intercept of linear regime of M–S plot. The fitting result shows a larger *V*_bi_ for the HI-manipulated CsPbI_3_ QDs compared with the control device (1.16 V), which follows a similar trend to the *V*_OC_ measured from the *J–V* curves. The enhanced *V*_bi_ could be attributed the reduction in non-radiative recombination through this passivation technique [54]. Moreover, thermal admittance spectroscopy analysis was carried out to obtain insight into the energetic distribution of trap density of states (tDOS). As shown in Fig. 4g, the tDOS with an energy level above 0.40 eV (area II and area III) decreased slightly with an obvious drop in the energy region between 0.30 and 0.40 eV (area I), indicating the effective passivation of shallower traps states, i.e., iodine-vacancy dominated shallow level defects [55, 56]. In addition, as shown in Fig. 4h, the best performing control and HI-manipulated devices exhibit a minimal trap states of 2.60 × 10^16^ and 1.69 × 10^16^ cm^−3^, respectively. These results evidently confirm that the HI-manipulated CsPbI_3_ QD device exhibits improved charge transport and reduced recombination, contributing to the prominent enhancement of *J*_sc_, and thus delivering overall enhanced performance of the CsPbI_3_ QD PV device.

## Conclusion

In summary, we developed a facile yet effective in situ passivation strategy of CsPbI_3_ to acquire high-quality CsPbI_3_ QDs. We revealed that a high I^−^ concentration is able to trigger highly coordinated polyiodide colloids from PbI_3_^−^ to PbI_4_^2−^ and PbI_5_^3−^, leading to higher crystallization and phase purity. We also demonstrated that the HI-manipulated CsPbI_3_ QDs possess a reduction in non-radiative recombination and near-unity PLQY by filling in surface iodine vacancies. Benefitting from the effective surface defects manipulation through the optimized feeding volume of HI, we obtained the champion PCE of 15.72% together with an enhanced storage stability. Our study provides fundamental insights into the nano-synthesis reaction process and blazes a new trail of controlling the nucleation and growth kinetics of CsPbI_3_ QDs. We highly expect that the present synthetic method can be extended to the regulations of other nanomaterials for high-performance optoelectronic applications.

### Supplementary Information

Below is the link to the electronic supplementary material.

Supplementary file1 (PDF 970 KB)
