# Supplementary material for: In Situ Iodide Passivation Toward Efficient CsPbI3 Perovskite Quantum Dot Solar Cells
Source: Nanomicro Lett. 2023 Jun 29;15:163. doi: 10.1007/s40820-023-01134-1 (PMC10310659; doi:10.1007/s40820-023-01134-1)
Supplement: Supplementary file 1 — Supplementary file1 (PDF 970 KB) [file 40820_2023_1134_MOESM1_ESM.pdf]

Supporting Information for

In-Situ Iodide Passivation Towards Efficient $CsPbI_3$ Perovskite Quantum Dot Solar Cells

Junwei Shi[1, 2], Ben Cohen-Kleinstein[3], Xuliang Zhang[2], Chenyu Zhao[2], Yong Zhang[1], Xufeng Ling[2], Junjun Guo[2], Doo-Hyun Ko[4], Baomin Xu[1, #], Jianyu Yuan[2, 5, #], Wanli Ma[2, 6, #]

[1]Department of Materials Science and Engineering, Southern University of Science and Technology, Shenzhen 518055, P. R. China

[2]Institute of Functional Nano & Soft Materials (FUNSOM), Soochow University, 199 Ren-Ai Road, Suzhou Industrial Park, Suzhou 215123, P. R. China

[3]Department of Electrical and Computer Engineering, University of British Columbia, 2329 West Mall, Vancouver, BC V6T 1Z4, Canada

[4]Department of Chemistry, Sungkyunkwan University, Suwon 16419, Republic of Korea

[5]Jiangsu Key Laboratory of Advanced Negative Carbon Technologies, Soochow University, 199 Ren-Ai Road, Suzhou Industrial Park, Suzhou 215123, P. R. China

[6]Jiangsu Key Laboratory for Carbon-Based Functional Materials & Devices, Soochow University, 199 Ren-Ai Road, Suzhou Industrial Park, Suzhou 215123, P. R. China

[#]Corresponding authors. E-mail: xubm@sustech.edu.cn (B. X.); jyyuan@suda.edu.cn (J. Y.); wlma@suda.edu.cn (W. M.)

Supplementary Figures and Table

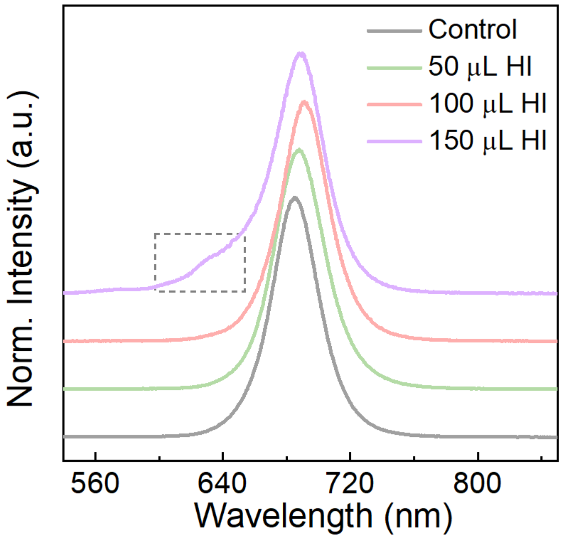

Fig. S1 PL emission spectra of $CsPbI_3$ QDs with various feeding volume of HI

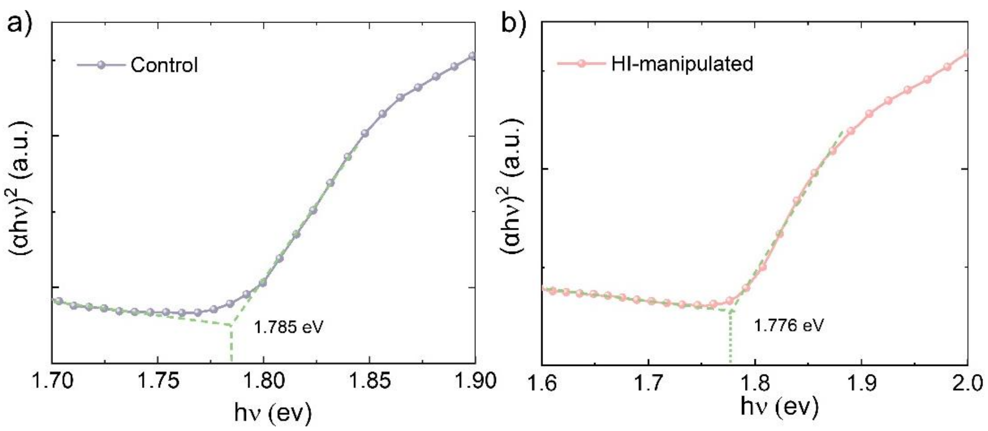

Fig. S2 Tauc plots of absorbance with photon energy (*hv*) and extracted bandgaps of control (1.785 eV) and HI-manipulated (1.776 eV) $CsPbI_3$ QDs

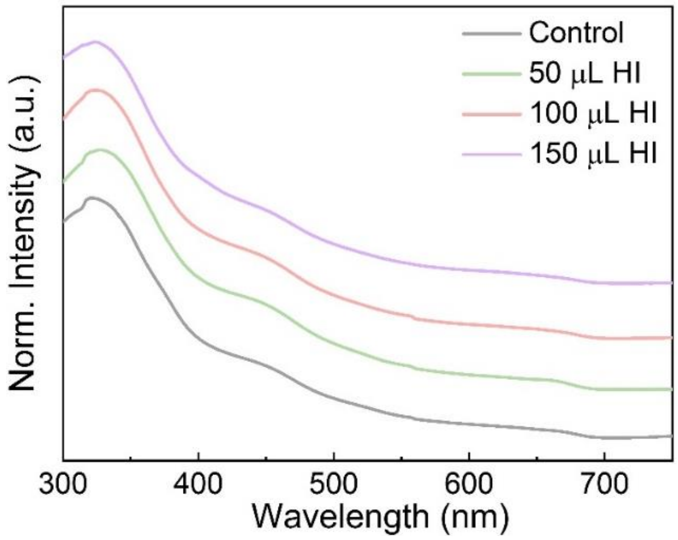

Fig. S3 UV-vis absorption of control and HI manipulated $CsPbI_3$ QDs

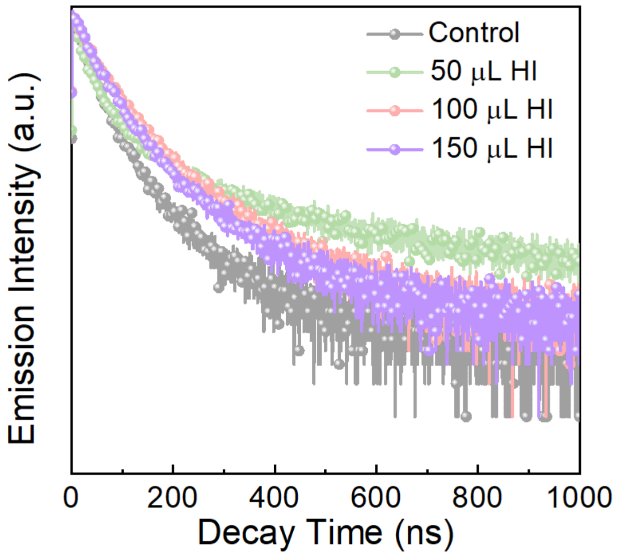

Fig. S4 TRPL decay curves of $CsPbI_3$ QDs with various feeding volume of HI

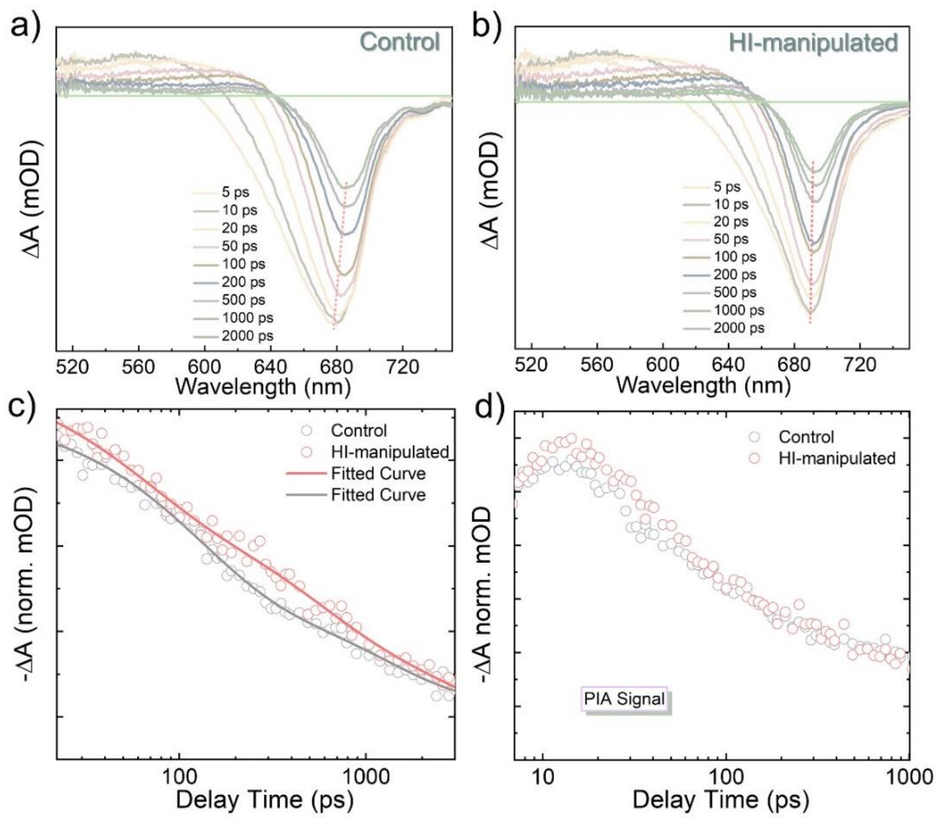

Fig. S5 a, b TAs spectra and **c, d** the extracted decay curves of control and HI-manipulated $CsPbI_3$ QDs

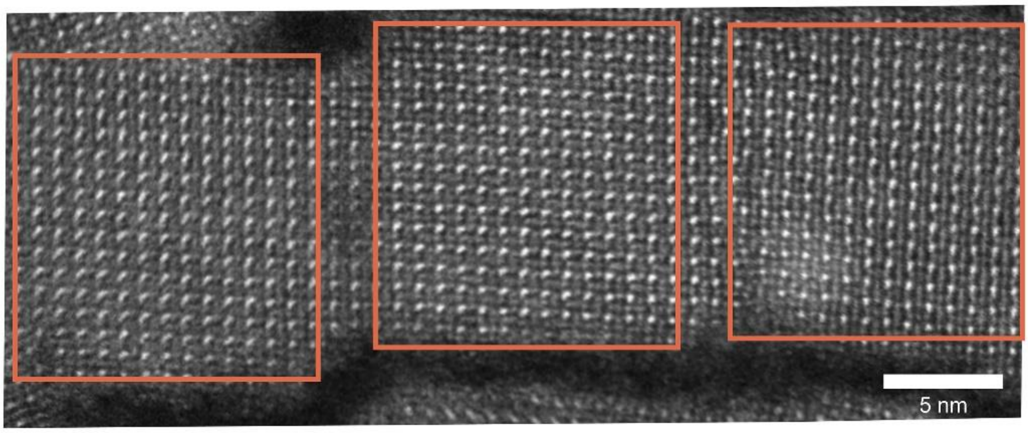

Fig. S6 The spherical aberration-corrected TEM captured picture of crystalline grain fusion- expansion along the direction of the (100) crystallographic plane

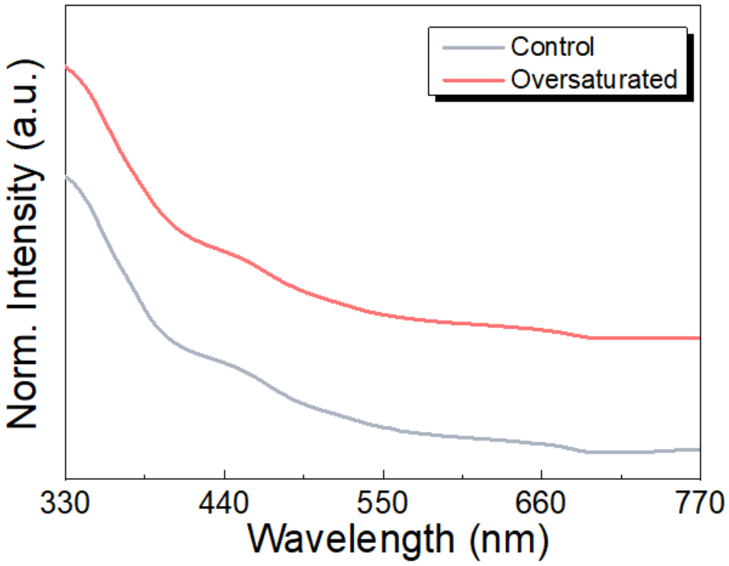

Fig. S7 UV-vis absorption of control and oversaturated HI feeding volume manipulated $CsPbI_3$ QDs

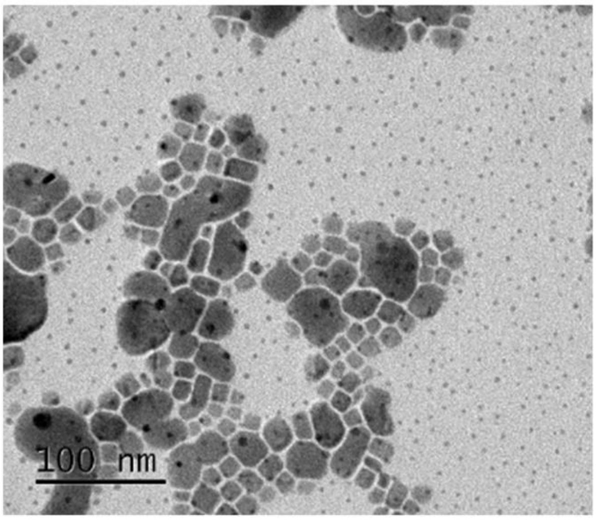

Fig. S8 TEM image of overloading HI-manipulated $CsPbI_3$ QDs

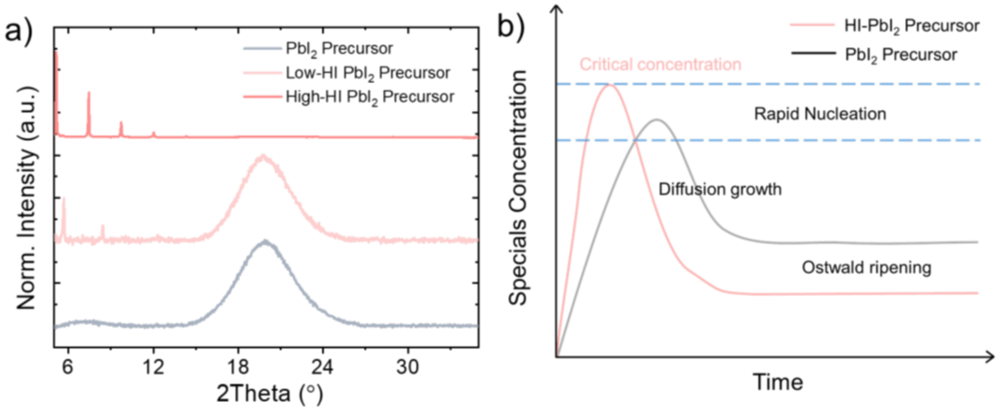

Fig. S9 a) XRD pattern of PbI_2-OA-OLA precursor w/wo the addition of HI. **b)** The simulated lamer model of $CsPbI_3$ QDs enucleation and growth dynamics process [S1]

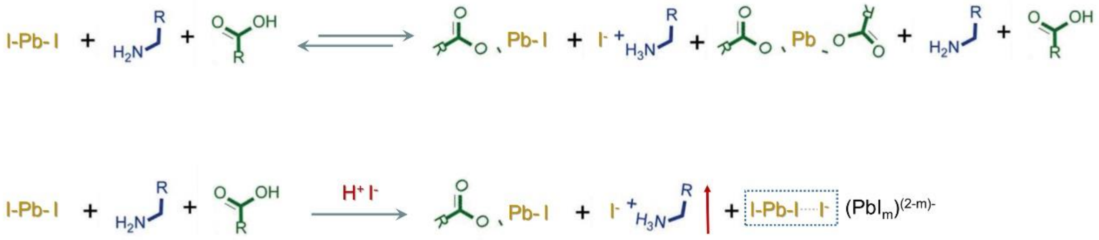

Fig. S10 The dynamics process of existing specials in Pb-I-precursor with or without HI manipulation

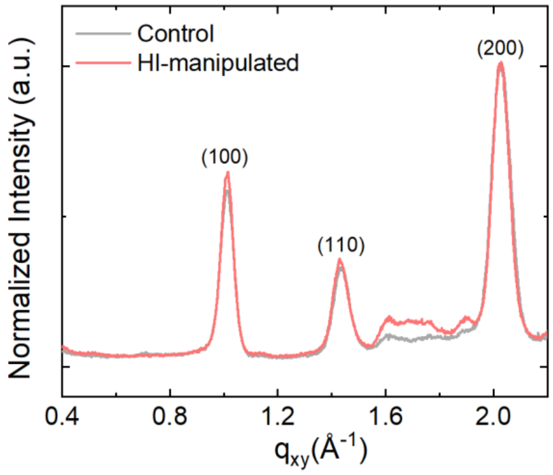

Fig. S11 The extracted diffraction curves of control and HI-manipulated $CsPbI_3$ QD films from GIWAX patterns

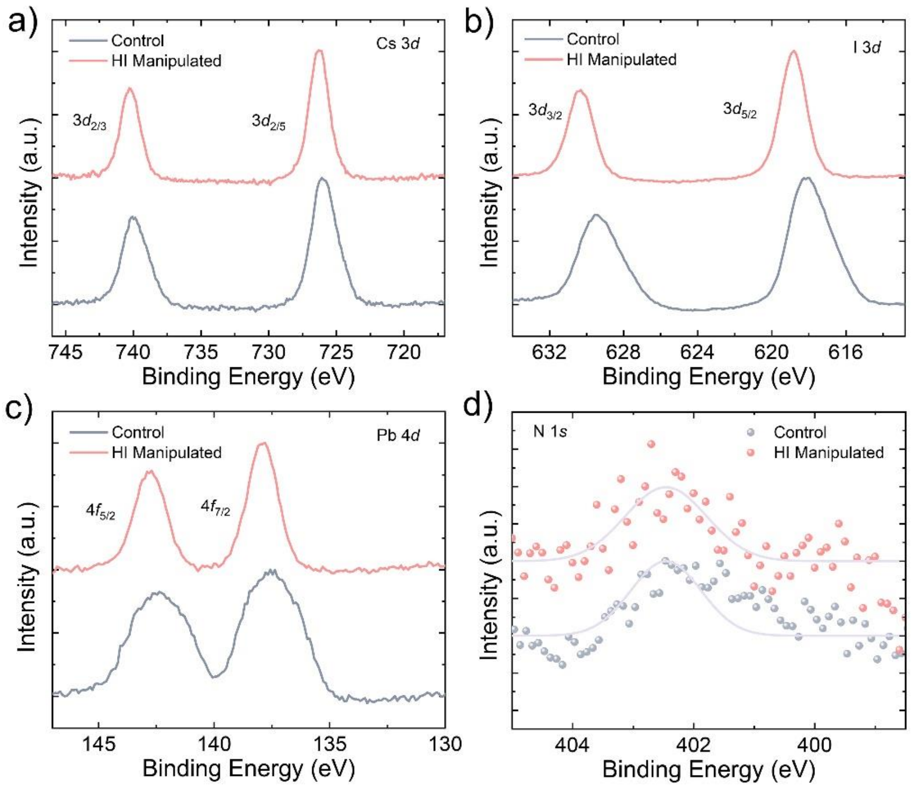

Fig. S12 XPS core level spectra of **a)** Cs *3d*, **b)** I *3d*, **c)** Pb *4f*, **d)** N 1s

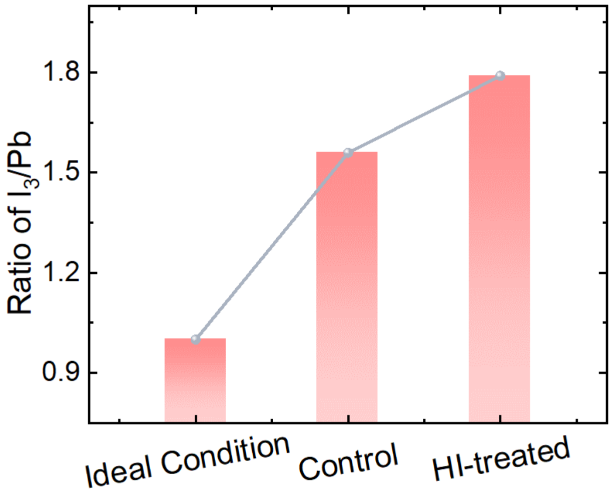

Fig. S13 I_3/Pb ratio of control and optimal HI-manipulated $CsPbI_3$ QDs

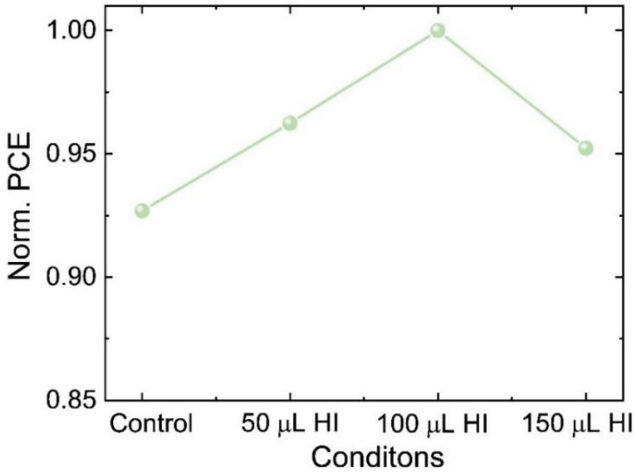

Fig. S14 The normalized device performance of $CsPbI_3$ QD solar cells under various conditions

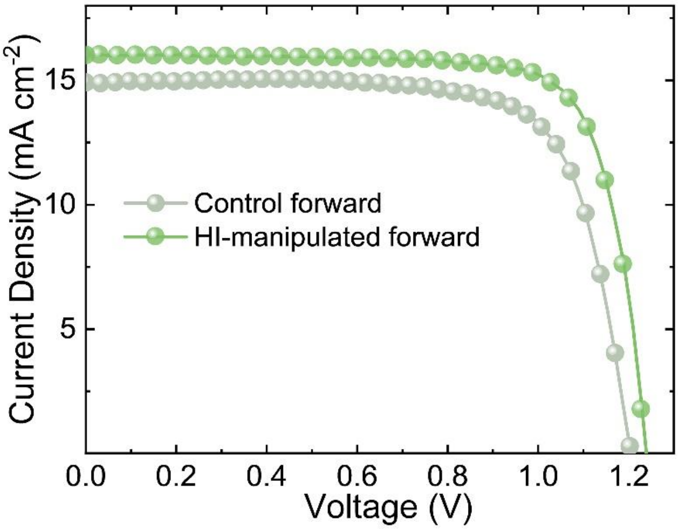

Fig. S15 *J*-*V* curves of the control and HI-manipulated devices under the forward scan direction

Table S1 The fitted parameters of TAs.

	A_1 (%)	τ_1	A_2 (%)	τ_2	A_3 (%)	τ_3 (ns)
Reference	31.97	118.69	33.02	118.69	35.01	1270.11
HI-manipulated	37.76	54.275	33.74	458.54	28.50	2839.82

Table S2 The detailed parameters of decay amplitude and average decay time fitted from TRPL

	τ_{ave}	A_1	τ_1	A_2	τ_2
Reference	35.74 ns	72.87%	28.83 ns	27.13%	100.38 ns
50 µl HI	44.63 ns	58.18%	36.85 ns	41.82%	114.22ns
100 µl HI	58.51 ns	64.75%	43.97 ns	35.25%	148.99 ns
150 µl HI	52.33 ns	53.23%	92.25 ns	46.77%	35.05 ns

Table S3 The PLQY values of $CsPbI_3$ QD solution with various HI loadings

	Control	50 µl HI	100 µl HI	150 µl HI
PL QY values	74%	92%	94%	90%

Table S4 PV device parameters extracted from *J-V* scans of control and HI-manipulated devices

Condition	Scan direction	V_{oc} (V)	J_{sc} (mA cm^{-2})	*FF* (%)	PCE (%)	*H*-index [a)]
Control	Reverse	1.21	15.30	76.01	14.07	5.47
	Forward	1.20	14.93	74.22	13.30	
HI-manipulated	Reverse	1.25	16.25	77.39	15.72	2.10
	Forward	1.24	16.03	77.47	15.39	

a) Hysteresis index, denoted as H-index, defined as $\mathrm{H} = \frac{PCE_{\mathrm{Rev}} - PCE_{For}}{PCE_{Rev}} \times 100$.

Table S5 The extracted EIS parameters of control and HI-manipulated devices

	R_s (Ohm)	R_{rec}(Ohm)	C_{rec}(F)
Control	109.60	18140	6.78×10^{-9}
HI-manipulated	93.05	33360	6.42×10^{-9}

Supplementary Reference

[S1]Z. Long, M. Liu, X.-g. Wu, K. Gu, G. Yang, Z. Chen, Y. Liu, R. Liu, H. Zhong. A reactivity-controlled epitaxial growth strategy for synthesizing large nanocrystals. Nat. Synth. **2,** 296–304 (2023). https://doi.org/10.1038/s44160-022-00210-5
